# Treating children with attention-deficit/hyperactivity disorder and comorbid epilepsy

**DOI:** 10.4103/0972-2327.78042

**Published:** 2011

**Authors:** Shivanand Kattimani, S Mahadevan

**Affiliations:** Department of Psychiatry, Jawaharlal Institute of Postgraduate Medical Education and Research (JIPMER), Pondicherry, India; 1 Department of Pediatrics, Jawaharlal Institute of Postgraduate Medical Education and Research (JIPMER), Pondicherry, India

**Keywords:** Attention-deficit/hyperactivity disorder, epilepsy, treatment

## Abstract

Attention-deficit/hyperactivity disorder (ADHD) is one of common neurodevelopmental disorder often comorbid with epilepsy. There are no existing guidelines on how to manage these two conditions when they are comorbid. To identify relationship between epilepsy and ADHD and to know role of antiepileptics and safety of stimulant like methylphenidate in such conditions from existing literature, we searched articles published in clinical journals available online between 1990-2010, with these key words in medline:children, epilepsy, seizure, comorbid, ADHD, treatment. Relevant abstracts were further selected for their focus on current topic. Cross references were extracted. Finally relevant articles that included original research articles, reviews and abstracts of non-english literature were used. Children with epilepsy may manifest with symptoms of ADHD. Children with ADHD may develop epilepsy. Some antiepileptics like phenobarbitone, gabapentin, topiramate may not be helpful in controlling behavioral symptoms of ADHD. Stimulants are the main stay of pharmacotherapy for ADHD but there is risk of decreasing seizure threshold in children with comorbid epilepsy especially when their epilepsy is not well controlled. Existing evidence is not in favor of screening children with ADHD for EEG abnormality before starting stimulant therapy.

## Introduction

Attention-deficit/hyperactivity disorder (ADHD) is a common neurodevelopmental disorder of childhood onset. It is being recognized for its impact on child development and on the family. This disorder is being treated by various health professionals, including pediatricians, psychiatrists, neurologists, internists, and general practitioners. Commonly used medications are stimulants, such as methylphenidate, amphetamine, and the nonstimulant drug atomoxetine. Carbamazepine and clonidine are used as non-FDA approved offlabel medications for ADHD in children and adolescents as an alternative to stimulants.[1,2] Besides other comorbid disorders, epilepsy is commonly encountered in children with ADHD. Treatment of ADHD with stimulants, such as methylphenidate, may not induce seizures but requires caution in those with recent epileptic seizures. Most controlled clinical drug trials using stimulants have excluded children having history of epileptic seizure because a theoretical risk of seizure exacerbation exists while using stimulants. One study that used a stimulant for treating ADHD symptoms in children with comorbid epilepsy has noticed increased seizure risk with increasing dose of the stimulant compared with placebo.[3] There are no guidelines on how to manage children with diagnosis of both ADHD and epilepsy. The American Association of Child and Adolescent Psychiatry (AACAP) and the American Association of Pediatrics (AAP) have issued guidelines on managing children with ADHD but have not addressed the current topic of dilemma.[4,5]

Children with epilepsy are likely to have higher lifetime prevalence of depressive disorders, anxiety, and ADHD compared with those without epilepsy.[6] The prevalence of ADHD in children in the general population is 5%–7%, whereas in children with epilepsy it is 20%–40%.[7,8] Inattention is more common than hyperactivity and impulsivity as a form of ADHD symptom in children with epilepsy.[7,9] Several factors contribute to the comorbidity of ADHD with epilepsy, including the underlying brain pathology, genetic predisposition, noradrenergic system dysregulation, chronic effects of seizures, effects of antiepileptic drugs, effects of stimulants, and psychosocial factors.[10]

Epilepsy in children is associated with behavioral problems, such as ADHD and cognitive impairments. Children with benign childhood epilepsy with centrotemporal spikes (BCECTS), complex partial seizures (CPS), frontal lobe epilepsy, rolandic epilepsy, and absence type of epileptic seizure may be more likely to show such symptoms.[8,11,12] Cognitive abilities are more likely to worsen if the age of onset of epileptic seizures is very early and epilepsy is uncontrolled.[11] Treatment with antiepileptics halts cognitive deterioration and improves attention and behavior,[12,13] and antiepileptics do not seem to have adverse effects on cognitive development of the children.[8] However, there are certain antiepileptics that adversely affect the attention and behavior even when used within their therapeutic range, for example, phenobarbitone, gabapentin, and topiramate.[14]

New onset epileptic seizures lead to cognitive deterioration and certain epileptic seizures, such as CPS may cause more inattention problems in children with ADHD.[15,16] In such cases, control of epileptic seizures from recurring improves attentional problems and further improvement can be derived from stimulant treatment. Sometimes inattention is difficult to differentiate as to whether it is a part of ADHD or caused by epileptic seizure. This can be found with the help of neuropsychologic tests and video electroencephalogram (VEEG).[17,18]

Children with ADHD are predisposed to epileptic seizures.[9] Children with ADHD have a higher than normal rate of EEG abnormalities without a history of epilepsy.[8] Such epileptiform discharges are reported to be associated with cognitive impairment and manifestation of ADHD symptoms[19] and in such cases some have shown that antiepileptic use may abolish epileptiform discharges and even improve ADHD symptoms.[20] Theoretically, the treatment of ADHD with stimulants, such as methylphenidate, is likely to increase the risk for seizure by lowering seizure threshold.[21] This raises the question as to whether all children with ADHD even without a history of epilepsy need to undergo EEG investigation to decide whether addition of antiepileptics is required before starting methylphenidate.

Some studies have shown that methylphenidate and atomoxetine use in children with ADHD without a history of epileptic seizure is safe.[22,23] However, safety of use of methylphenidate in those with ADHD and comorbid epilepsy or those with abnormal EEG without a history of epilepsy in the short-term as well as in the long-term course remains to be ascertained.

The number of children enrolled varied from 10 to 119 in studies that have tried to ascertain this in short term course. The duration of stimulant exposure varied from 8 weeks to 10 months in these randomized controlled studies. Many have used placebo and some compared the effects of methylphenidate with dextroamphetamine. Some studies have reported no increased seizure frequency or any major side effects in ADHD children with comorbid epilepsy.[16,24–26]

Those who found increased seizure frequency while on methylphenidate have found different explanations: this happened (1) in those with uncontrolled epilepsy[28–29] and (2) in those with abnormal epileptiform discharges even in the absence of a history of epilepsy.[21] One point to be noted is that those children with uncontrolled epilepsy at risk for increased seizures with methylphenidate were on antiepileptics. Use of methylphenidate in those children with ADHD having comorbid epilepsy well controlled by antiepileptics was found not to have any effect on background EEG changes, seizure frequency, or anticonvulsant drug levels.[24]

However, the risk of epileptic seizure exists in those with history of epilepsy, having diseases involving the central nervous system, having metabolic disorders affecting the central nervous system, and being on psychotropics.[8,30]

There is still a need for large sample-sized, randomized controlled studies to collect data on the safety and efficacy of stimulants in children with ADHD and epilepsy. As there is insufficient evidence, EEG screening is not recommended in children presenting with ADHD unless there are other clinical indications. Although evidence at this time shows that use of methylphenidate for the treatment of ADHD does not alter antiepileptic drug levels or seizure frequency, still the longer-term effects of methylphenidate itself and its effects on children with frequent epileptic seizures need further study.

In cases where epilepsy and ADHD are comorbid, carbamazepine and lamotrigine may be more beneficial compared with other antiepileptics in that apart from the control of epileptic seizure they have better effects on attention and behavior.[14] If there is comorbid ADHD that can be diagnosed independent of epilepsy effects, then further improvement in inattention and hyperactive/impulsive symptoms can be brought about by the addition of a specific ADHD treatment. Although there are no recommended guidelines, we, the authors are of the opinion that if inattention and hyperactivity symptoms persist for more than 6 months after the last epileptic seizure, one can go for a stimulant medication trial.
